# Unusual case of human herpesvirus 8‐positive large B‐cell lymphoma associated with Castleman disease

**DOI:** 10.1002/ccr3.2015

**Published:** 2019-02-07

**Authors:** Narittee Sukswai, Kirill Lyapichev, L. Jeffrey Medeiros, Joseph D. Khoury

**Affiliations:** ^1^ Department of Hematopathology The University of Texas MD Anderson Cancer Center Houston Texas; ^2^ Faculty of Medicine, Department of Pathology Chulalongkorn University Bangkok Thailand

**Keywords:** Castleman disease, diffuse large B‐cell lymphoma, germinotropic lymphoproliferative disorder, human herpes virus 8

## Abstract

An overlap between human herpesvirus 8 (HHV8) ‐positive diffuse large B‐cell lymphoma and HHV8‐positive germinotropic lymphoproliferative disorder has been proposed. We present a unique Epstein‐Barr virus‐associated case in which features of both conditions were present.

A 56‐year‐old man with known well‐controlled human immunodeficiency virus (HIV) infection presented with tender and progressive right axillary lymphadenopathy without B symptoms. Imaging demonstrated enlarged cervical, axillary, and supraclavicular lymph nodes with avid uptake of 15‐fluorodeoxyglucose (Figure 1A). Excisional biopsy of a right axillary lymph node showed preserved nodal architecture with infiltrative neoplastic cells replacing germinal centers (Figure 1B) and infiltrating around mantle zones and into sinusoids (Figure 1C). Residual lymphoid follicles were atrophic, had hyaline‐vascular changes, “onion skin” mantle zones, and prominent high endothelial venules (Figure 1D). Neoplastic cells had plasmablastic morphology, with vesicular eccentric nuclei, prominent nucleoli, and abundant amphophilic cytoplasm (Figure 1E). They were positive for human herpesvirus 8 (HHV8) (Figure 1F), Epstein‐Barr virus (EBV) (Figure 1G), MUM1/IRF4 (Figure 1H), CD20 (focal, weak), and Myc overexpression, without CD30, CD138, kappa or lambda expression. Since the neoplastic cells infiltrated beyond germinal centers, a diagnosis of HHV8‐positive diffuse large B‐cell lymphoma (HHV8+ DLBCL) associated with Castleman disease was rendered. HHV8‐associated lymphoproliferative disorders include multicentric Castleman disease, HHV8+ DLBCL (EBV‐negative usually), and HHV8+ germinotropic lymphoproliferative disorder (GLPD) (EBV‐positive usually). GLPD patients are asymptomatic and have a favorable response to chemotherapy or radiation, whereas HHV8+ DLBCL is associated with poor prognosis.^1^, ^2^ This unusual case provides further evidence of overlap between HHV8+ DLBCL and GLPD.

**Figure 1:**
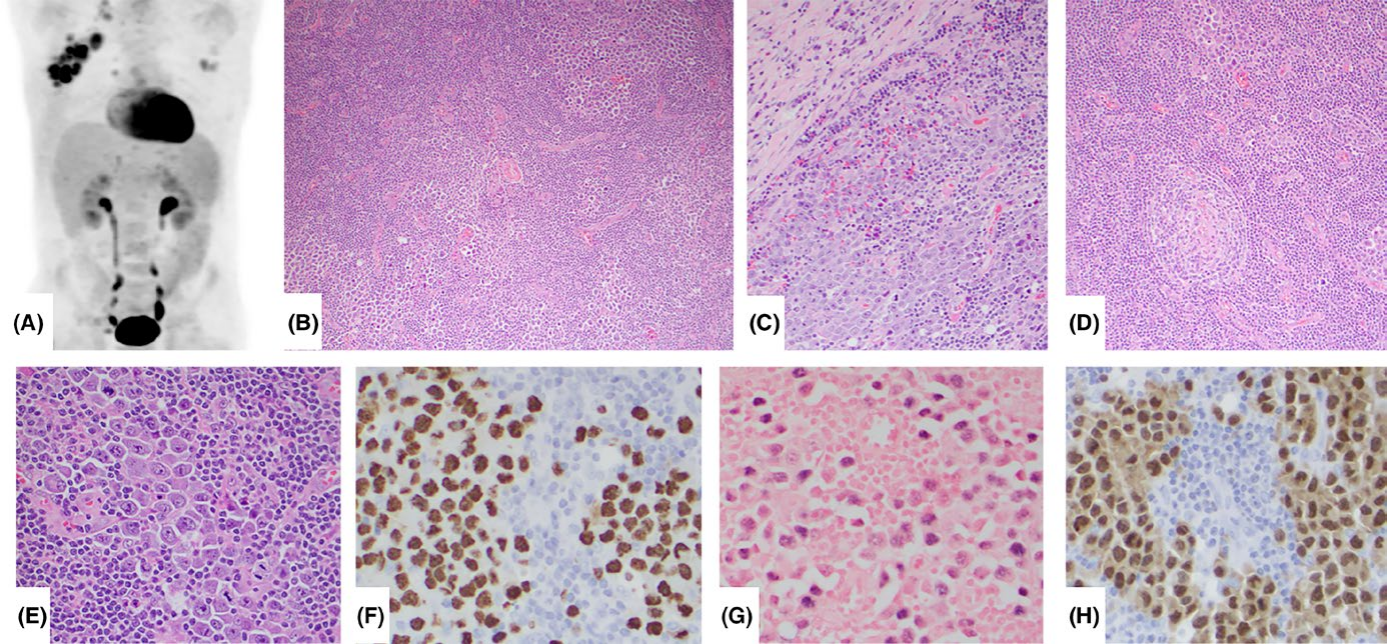
15‐fluorodeoxyglucose (FDG) positron emission tomography scan demonstrated multiple enlarged FDG‐avid regional lymph nodes (A). Lymph node excisional biopsy revealed multiple foci of neoplastic cells replacing some germinal centers (B). These cells were also present in mantle zones surrounding benign germinal centers, and other clusters were noted within sinusoids (C). Most residual lymphoid follicles were atretic and had hyaline‐vascular changes, “onion skin” mantle zones, and prominent interfollicular high endothelial venules (D). The neoplastic cells had plasmablastic morphology, characterized by large cells with vesicular eccentric nuclei, prominent nucleoli and abundant amphophilic cytoplasm (E). The neoplastic cells were positive for human herpesvirus 8 (HHV‐8) (F), Epstein‐Barr virus‐encoded RNA (G), and MUM1/IRF4 (H)

## CONFLICT OF INTEREST

None declared.

## AUTHOR CONTRIBUTION

NS: participated in manuscript writing, collection of images, and literature review. KL: participated in manuscript review. LJM: participated in manuscript review. JDK: participated in manuscript review.
